# On ***C*_*ℵ*_**-Fibrations in Bitopological Semigroups

**DOI:** 10.1155/2014/675761

**Published:** 2014-08-17

**Authors:** Suliman Dawood, Adem Kılıçman

**Affiliations:** ^1^Department of Mathematical Sciences, Hodeidah University, Hodeidah, Yemen; ^2^Department of Mathematics and Institute for Mathematical Research, University Putra Malaysia (UPM), 43400 Serdang, Selangor, Malaysia

## Abstract

We extend the path lifting property in homotopy theory for topological spaces to bitopological semigroups and we show and prove its role in the *C*_*ℵ*_-fibration property. We give and prove the relationship between the *C*_*ℵ*_-fibration property and an approximate fibration property. Furthermore, we study the pullback maps for *C*_*ℵ*_-fibrations.

## 1. Introduction

In homotopy theory for topological space (i.e., spaces), Hurewicz [1] introduced the concepts of fibrations and path lifting property of maps and showed its equivalence with the covering homotopy property. Coram and Duvall [2] introduced approximate fibrations as a generalization of cell-like maps [3] and showed that the uniform limit of a sequence of Hurewicz fibrations is an approximate fibration. In 1963, Kelly [4] introduced the notion of bitopological spaces. Such spaces were equipped with its two (arbitrary) topologies. The reader is suggested to refer to [4] for the detail definitions and notations. The concept of homotopy theory for topological semigroups has been introduced by Cerin in 2002 [5]. In this theory, he introduced *S*_*ℵ*_-fibrations as extension of Hurewicz fibrations. In [6], we introduced the concepts of bitopological semigroups, *c*-bitopological semigroups, and *C*_*ℵ*_-fibrations as extension of *S*_*ℵ*_-fibrations.

This paper is organized as follows. It consists of five sections. After this Introduction, Section 2 is devoted to some preliminaries. In Section 3 we show the pullbacks of *S*-maps which have the *C*_*ℵ*_-fibration property that will also have this property and the pullbacks of *C*_*ℵ*_-fibrations are *C*_*ℵ*_-fibrations under given conditions. In Section 4 we develop and extend path lifting property in homotopy theory for topological semigroups to theory for bitopological semigroups. Some results about Hurewicz fibrations carry over. In Section 5 we give and prove the relationship between the *C*_*N*_*π*__-fibration property and an approximate fibration property.

## 2. Preliminaries

Throughout this paper, by all *X*_*τ*_ we mean all topological spaces (*X*, *τ*) which will be assumed Hausdorff spaces. By all *X*_*τ*_12__ we mean all bitopological spaces (*X*, *τ*_1_, *τ*_2_). For two bitopological spaces *X*_*τ*_12__ and *Y*_*ρ*_12__, a* p-map h* : *X*_*τ*_12__ → *Y*_*ρ*_12__ is a function from *X* into *Y* that is continuous function (i.e., a map) from a space *X*_*τ*_1__ into a space *Y*_*ρ*_1__ and from *X*_*τ*_2__ into *Y*_*ρ*_2__ [4].

Recall [5] that a* topological semigroup* or an* S-space* is a pair (*X*_*τ*_, ∗) consisting of a topological space *X*_*τ*_ and a map ∗ : *X*_*τ*_ × *X*_*τ*_ → *X*_*τ*_ from the product space *X*_*τ*_ × *X*_*τ*_ into *X*_*τ*_ such that ∗(*x*, ∗(*y*, *z*)) = ∗(∗(*x*, *y*), *z*) for all *x*, *y*, *z* ∈ *X*. An* S*-space (*A*, ∗′) is called an* S-subspace* of (*X*_*τ*_, ∗) if *A* is a subspace of *X*_*τ*_ and the map ∗ takes the product *A* × *A* into *A* and ∗′(*x*, *y*) = ∗(*x*, *y*) for all *x*, *y* ∈ *A*. We denote the class of all* S*-spaces by *ℵ*. For every space *X*_*τ*_, by *P*(*X*_*τ*_), we mean the space of all paths from the unit closed interval *I* = [0,1] into *X*_*τ*_ with the compact-open topology. Recall [5] that, for every* S*-space (*X*_*τ*_, ∗), (*P*(*X*_*τ*_), *p*(∗)) is an* S*-space where *p*(∗) : *P*(*X*_*τ*_) × *P*(*X*_*τ*_) → *P*(*X*_*τ*_) is a map defined by *p*(∗)(*α*, *β*)(*t*) = ∗(*α*(*t*), *β*(*t*)) for all *α*, *β* ∈ *P*(*X*_*τ*_), *t* ∈ *I*. The shorter notion for this* S*-space will be *P*(*X*_*τ*_, ∗). For every space *X*_*τ*_, the* natural S-space* is an* S*-space (*X*_*τ*_, *π*_*i*_), where *π*_*i*_ is a continuous associative multiplication on *X*_*τ*_ given by *π*_1_(*x*, *y*) = *x* and *π*_2_(*x*, *y*) = *y* for all *x*, *y* ∈ *X*. We denote the class of all natural* S*-spaces (*X*_*τ*_, *π*) by *N*_*π*_, where *π* = *π*_1_, *π*_2_.

Recall [5] that the function *f* : (*X*_*τ*_, ∗)→(*O*_*ρ*_, ∘) is called an* S-map* if *f* is a map of a space *X*_*τ*_ into *O*_*ρ*_ and *f*(∗(*x*, *y*)) = ∘(*f*(*x*), *f*(*y*)) for all *x*, *y* ∈ *X*. The function *f* : *X*_*τ*_ → *O*_*ρ*_ of a natural* S*-space (*X*_*τ*_, *π*) into (*O*_*ρ*_, *π*) is an* S*-map if and only if it is continuous. The* S*-maps *f*, *g* : (*X*_*τ*_, ∗)→(*O*_*ρ*_, ∘) are called* S-homotopic* and write *f* ≃_*s*_ *g* provided there is an* S*-map *H* : (*X*_*τ*_, ∗) → *P*(*O*_*ρ*_, ∘) called an* S-homotopy* such that *H*(*x*)(0) = *f*(*x*) and *H*(*x*)(1) = *g*(*x*) for all *x* ∈ *X*.

A* bitopological semigroup* is a pair (*X*_*τ*_12__, ∗) consisting of a bitopological space *X*_*τ*_12__ and the associative multiplication ∗ on *X* such that ∗ is an *p*-map from the product bitopological space (*X* × *X*, *τ*_1_ × *τ*_1_, *τ*_2_ × *τ*_2_) into *X*_*τ*_12__. For *B*⊆*X*, by *B*|_*τ*_12__ we mean the bitopological subspace (*B*, *τ*_1_|_*B*_, *τ*_2_|_*B*_) of *X*_*τ*_12__. If the *p*-map ∗ takes the product *B* × *B* into *B* then the pair (*B*|_*τ*_12__, ∗) will be a bitopological semigroup and will be called an* b-subspace* of (*X*_*τ*_12__, ∗).

The function *h* : (*X*_*τ*_12__, ∗)→(*Y*_*ρ*_12__, ∘) is called an *S*_*i*_-*map* from (*X*_*τ*_12__, ∗) into (*Y*_*ρ*_12__, ∘) provided *h* is an* S*-map from a function* S*-space (*X*_*τ*_*i*__, ∗) into an* S*-space (*Y*_*ρ*_*i*__, ∘), where *i* = 1,2. We say that *h* is an* Sp-map* if it is an *S*_1_-map and *S*_2_-map.

An* c-bitopological semigroup* is a triple (*X*_*τ*_12__, ∗, *X*) consisting of bitopological semigroups (*X*_*τ*_12__, ∗) and an* S*-map *X* : (*X*_*τ*_2__, ∗)→(*X*_*τ*_1__, ∗) from an* S*-space (*X*_*τ*_2__, ∗) into an* S*-space (*X*_*τ*_1__, ∗). In our work, for any* S*-space, (*O*_*ρ*_, ∘) can be regarded as an *c*-bitopological semigroup (*O*_*ρρ*_, ∘, id) where id is the identity* S*-map on (*O*_*ρ*_, ∘). That is, (*O*_*ρ*_, ∘): = (*O*_*ρρ*_, ∘, id).

An* c-map* from (*X*_*τ*_12__, ∗, *X*) into (*O*_*ρ*_, ∘) is a pair *f*_12_ = (*f*_1_, *f*_2_):(*X*_*τ*_12__, ∗, *X*)→(*O*_*ρ*_, ∘) of an *S*_1_-map *f*_1_ : (*X*_*τ*_1__, ∗)→(*O*_*ρ*_, ∘) and *S*_2_-map *f*_2_ : (*X*_*τ*_2__, ∗)→(*O*_*ρ*_, ∘) such that *f*_1_∘*X* = *f*_2_.

**Definition 1 (see [6]).**

Let *f* : (*X*_*τ*_, ∗)→(*O*_*ρ*_, ∘) and *h* : (*X*_*τ*′_′, ∗′)→(*X*_*τ*_, ∗) be two* S*-maps. An* S*-map *f* is said to have the *C*_*ℵ*_-fibration property by an* S*-map *h* provided for every (*Y*_*ω*_, ⋆) ∈ *ℵ* and, given two* S*-maps *g* : (*Y*_*ω*_, ⋆)→(*X*_*τ*′_′, ∗′) and *G* : (*Y*_*ω*_, ⋆) → *P*(*O*_*ρ*_, ∘) with *G*_0_ = *f*∘(*h*∘*g*), there exists an* S*-homotopy *H* : (*Y*_*ω*_, ⋆) → *P*(*X*_*τ*_, ∗) such that *H*_0_ = *h*∘*g* and *f*∘*H*_*t*_ = *G*_*t*_ for all *t* ∈ *I*.

**Definition 2 (see [6]).**

An *c*-map *f*_12_ : (*X*_*τ*_12__, ∗, *X*)→(*O*_*ρ*_, ∘) is called an *C*_*ℵ*_-fibration if an *S*_1_-map *f*_1_ : (*X*_*τ*_1__, ∗)→(*O*_*ρ*_, ∘) has the *C*_*ℵ*_-fibration property by an* S*-map *X* : (*X*_*τ*_2__, ∗)→(*X*_*τ*_1__, ∗). That is, for every (*Y*_*ω*_, ⋆) ∈ *ℵ* and given two* S*-maps *g* : (*Y*_*ω*_, ⋆)→(*X*_*τ*_2__, ∗) and *G* : (*Y*_*ω*_, ⋆) → *P*(*O*_*ρ*_, ∘) with *G*_0_ = *f*_2_∘*g*, there exists an* S*-homotopy *H* : (*Y*_*ω*_, ⋆) → *P*(*X*_*τ*_1__, ∗) such that *H*_0_ = *X*∘*g* and *f*_1_∘*H*_*t*_ = *G*_*t*_ for all *t* ∈ *I*.

Let (*X*_*τ*_12__, ∗, *B*) be an c-bitopological semigroup and let (*B*|_*τ*_12__, ∗) be an *b*-subspace of (*X*_*τ*_12__, ∗). The *c*-bitopological semigroup (*B*|_*τ*_12__, ∗, *B*) is called an* c-subspace* of (*X*_*τ*_12__, ∗, *X*) provided *B*(*b*) = *X*(*b*) for all *b* ∈ *B*.

**Theorem 3 (see [6]).**

Let *f*_12_ : (*X*_*τ*_12__, ∗, *X*)→(*O*_*ρ*_, ∘) be an c-map and (*B*|_*ρ*_, ∘) be an* S*-subspace of (*O*_*ρ*_, ∘) such that *f*_1_^−1^(*B*) = *f*_2_^−1^(*B*). Then the triple (*B* ^−^|_*τ*_12__, ∗, *X*|_*B*^−^_) is an c-subspace of (*X*_*τ*_12__, ∗, *X*) and a pair *f*_12_|_*B*^−^_ = (*f*_1_|_*B*^−^_, *f*_2_|_*B*^−^_) is an c-map from an c-bitopological semigroup (*B* ^−^|_*τ*_12__, ∗, *X*|_*B*^−^_) into (*B*|_*ρ*_, ∘), where *B* ^−^ = *f*_1_^−1^(*B*).

**Corollary 4 (see [6]).**

Let *f*_12_ : (*X*_*τ*_12__, ∗, *X*)→(*O*_*ρ*_, ∘) be an *C*_*ℵ*_-fibration and let (*B*|_*ρ*_, ∘) be an* S*-subspace of (*O*_*ρ*_, ∘) such that *f*_1_^−1^(*B*) = *f*_2_^−1^(*B*). Then the restriction c-map

(1) $$\begin{array}{c} {f_{12}|}_{B^{-}}:\left({B^{-}|}_{\tau_{12}},*,{X|}_{B^{-}}\right)⟶\left({B|}_{\rho },\circ \right) \end{array}$$

is an *C*_*ℵ*_-fibration, where *B* ^−^ = *f*_1_^−1^(*B*).

## 3. The Pullback c-Maps

In this section, we show that the pullbacks of* S*-maps which have the *C*_*ℵ*_-fibration property will also have this property and the pullbacks of *C*_*ℵ*_-fibrations are *C*_*ℵ*_-fibrations under given conditions.

Let *f*_12_ : (*X*_*τ*_12__, ∗, *X*)→(*O*_*ρ*_, ∘) be an *c*-map and let *h* : (*Q*_*υ*_, ⊙)→(*O*_*ρ*_, ∘) be an* S*-map. Let

(2) $$\begin{array}{c} X^{hi}=\left\{\left(x,b\right)\in X\times Q\;|\;f_{i}\left(x\right)=h\left(b\right)\right\}, \end{array}$$

where *i* = 1,2.

**Lemma 5 .**

Let *f*_12_ : (*X*_*τ*_12__, ∗, *X*)→(*O*_*ρ*_, ∘) be an c-map and let *h* : (*Q*_*υ*_, ⊙)→(*O*_*ρ*_, ⊙) be an* S*-map. Then the pair (*X* ^*hi*^|_*τ*_12_×*υ*_, ∗ × ⊙) is an b-subspace of the bitopological semigroup ((*X* × *Q*)_*τ*_12_×*υ*_, ∗ × ⊙), where *i* = 1,2.

**Proof**

It is clear that *X* ^*h*1^|_*τ*_12_×*υ*_ and *X* ^*h*2^|_*τ*_12_×*υ*_ are subspaces of a bitopological space (*X* × *O*)_*τ*_12_×*υ*_. Since *h* is an* S*-map and *f*_1_ is an *S*_1_-map, then, for all (*x*, *b*), (*x*′, *b*′) ∈ *X* ^*h*1^,

(3) $$\begin{array}{c} h\left(b⊙b^{\prime }\right)=h\left(b\right)\circ h\left(b^{\prime }\right)=f_{1}\left(x\right)\circ f_{1}\left(x^{\prime }\right)=f_{1}\left(x*x^{\prime }\right). \end{array}$$

This implies

(4) $$\begin{array}{c} \left(x,b\right)\left(*\times ⊙\right)\left(x^{\prime },b^{\prime }\right)=\left(x*x^{\prime },b⊙b^{\prime }\right)\in X^{h1} \end{array}$$

for all (*x*, *b*), (*x*′, *b*′) ∈ *X* ^*h*1^. That is, (*X* ^*h*1^|_*τ*_12_×*υ*_, ∗ × ⊙) is an *b*-subspace of the bitopological semigroup ((*X* × *O*)_*τ*_12_×*υ*_, ∗ × ⊙). Similarly, (*X* ^*h*2^|_*τ*_12_×*υ*_, ∗ × ⊙) is an *b*-subspace of the bitopological semigroup ((*X* × *O*)_*τ*_12_×*υ*_, ∗ × ⊙).

Henceforth, in this paper, by *J*_1_ and *J*_2_, we mean the usual first and the second projection* S*-maps (or maps), respectively.

**Theorem 6 .**

Let *f*_12_ : (*X*_*τ*_12__, ∗, *X*)→(*O*_*ρ*_, ∘) be an *C*_*ℵ*_-fibration and let *h* : (*Q*_*υ*_, ⊙)→(*O*_*ρ*_, ∘) be an* S*-map. Then the* S*-map *f* ^*h*1^ : (*X* ^*h*1^|_*τ*_1_×*υ*_, ∗ × ⊙)→(*Q*_*υ*_, ⊙) has the *C*_*ℵ*_-fibration property by an* S*-map *X* ^*h*^ = *X* × *id*|_*X*^*h*2^_ such that *f* ^*h*1^(*x*, *b*) = *b* for all (*x*, *b*) ∈ *X* ^*h*1^.

**Proof**

Since *f*_12_ is an *c*-map then, for all (*x*, *b*) ∈ *X* ^*h*2^,

(5) $$\begin{array}{c} f_{1}\left(X\left(x\right)\right)=f_{2}\left(x\right)=h\left(b\right). \end{array}$$

That is, (*X*(*x*), *b*) ∈ *X* ^*h*1^ for all (*x*, *b*) ∈ *X* ^*h*2^. Hence, by the last lemma, *X* ^*h*^ is a well-defined* S*-map taking (*X* ^*h*2^|_*τ*_2_×*υ*_, ∗ × ⊙) into (*X* ^*h*1^|_*τ*_1_×*υ*_, ∗ × ⊙).

Now let (*Y*_*ω*_, ⋆) ∈ *ℵ* and let *g* : (*Y*_*ω*_, ⋆)→(*X* ^*h*2^|_*τ*_2_×*υ*_, ∗ × ⊙) and *G* : (*Y*_*ω*_, ⋆) → *P*(*Q*_*υ*_, ⊙) be two* S*-maps with *G*_0_ = *f* ^*h*1^∘(*X* ^*h*^∘*g*).

Take an* S*-map *g*′ = *J*_1_∘*g* : (*Y*_*ω*_, ⋆)→(*X*_*τ*_2__, ∗) and an* S*-homotopy

(6) $$\begin{array}{c} G^{\prime }=h\circ G:\left(Y_{\omega },⋆\right)⟶P\left(O_{\rho },\circ \right). \end{array}$$

We observe that

(7) G′(y)(0)=h[G(y)(0)]=h[(fh1∘Xh)(g(y))]=h{fh1[X(J1(g(y))),J2(g(y))]}=h[J2(g(y))]=f2[J1(g(y))]=f2(g′(y))

for all *y* ∈ *Y*. That is, *G*_0_′ = *f*_2_∘*g*′. Since *f*_12_ is an *C*_*ℵ*_-fibration, then there is an* S*-homotopy *H*′ : (*Y*_*ω*_, ⋆) → *P*(*X*_*τ*_1__, ∗) such that *H*_0_′ = *X*∘*g*′ and *f*_1_∘*H*_*t*_′ = *G*_*t*_′ for all *t* ∈ *I*.

Define an* S*-homotopy *H* : (*Y*_*ω*_, ⋆) → *P*(*X* ^*h*1^|_*τ*_1_×*υ*_, ∗ × ⊙) by

(8) $$\begin{array}{c} H\left(y\right)\left(t\right)=\left[H^{\prime }\left(y\right)\left(t\right),G\left(y\right)\left(t\right)\right] \end{array}$$

for all *y* ∈ *Y*, *t* ∈ *I*. We observe that *f* ^*h*1^∘*H*_*t*_ = *G*_*t*_ for all *t* ∈ *I* and

(9) H(y)(0)=[H′(y)(0),G(y)(0)]=[X(g′(y)),(fh1∘Xh)(g(y))]=[X(J1(g(y))),J2(g(y))]=Xh[J1(g(y)),J2(g(y))]=Xh(g(y))=(Xh∘g)(y)

for all *y* ∈ *Y*. That is, *H*_0_ = *X* ^*h*^∘*g*. Hence *f* ^*h*1^ has the *C*_*ℵ*_-fibration property by an* S*-map *X* ^*h*^.

In the last theorem, if *f*_1_ = *f*_2_ (i.e., *f*_12_ is an *Sp*-map), let *f* = *f*_1_ = *f*_2_; then

(10) $$\begin{array}{c} X^{h}=X\times id{|}_{X^{h}}:\left(X^{h}{|}_{\tau_{2}\times \upsilon },*\times ⊙\right)⟶\left(X^{h}{|}_{\tau_{1}\times \upsilon },*\times ⊙\right) \end{array}$$

is a well-defined* S*-map taking (*X* ^*h*^|_*τ*_2_×*υ*_, ∗ × ⊙) into (*X* ^*h*^|_*τ*_1_×*υ*_, ∗ × ⊙), where *X* ^*h*^ = *X* ^*h*1^ = *X* ^*h*2^. That is, the triple (*X* ^*h*^|_*τ*_12_×*υ*_, ∗ × ⊙, *X* ^*h*^) is an *c*-bitopological semigroup, called a* pullback c-bitopological semigroup* of (*X*_*τ*_12__, ∗, *X*) induced from *f*_12_ by *h*. The pair

(11) $$\begin{array}{c} f_{12}^{h}=\left(f^{h},f^{h}\right):{\left(X^{h},\tau_{1}\times \upsilon {|}_{X^{h}},\tau_{2}\times \upsilon {|}_{X^{h}}\right)}_{X^{h}}⟶\left(Q_{\upsilon },⊙\right) \end{array}$$

which is given by *f* ^*h*^(*x*, *b*) = *b* for all (*x*, *b*) ∈ *X* ^*h*^ is an *c*-map, called a* pullback c-map* of *f*_12_ induced by *h*. We observe that

(12) $$\begin{array}{c} \left(f^{h}\circ X^{h}\right)\left(x,b\right)=f^{h}\left(X\left(x\right),b\right)=b=f^{h}\left(x,b\right) \end{array}$$

for all (*x*, *b*) ∈ *X* ^*h*^.

**Theorem 7 .**

Let *f*_12_ = (*f*, *f*):(*X*_*τ*_12__, ∗, *X*)→(*O*_*ρ*_, ∘) be an *C*_*ℵ*_-fibration and let *h* : (*Q*_*υ*_, ⊙)→(*O*_*ρ*_, ∘) be an* S*-map such that *X* ^*h*1^∩*X* ^*h*2^ ≠ *ϕ*. Then the pullback *c*-map *f*_12_^*h*^ of *f*_12_ induced by *h* is an *C*_*ℵ*_-fibration.

**Proof**

It is obvious by the last theorem and the second part in Definition 2.

## 4. The c-Lifting Functions

In this section, we define the path lifting property for *c*-maps by giving the concept of an *c*-lifting property and we show its role in satisfying the *C*_*ℵ*_-fibration property.

Recall [5] that for an* S*-map *f* : (*X*_*τ*_, ∗)→(*O*_*ρ*_, ∘), the map: *α* → *f*∘*α* for all *α* ∈ *P*(*X*_*τ*_) is an* S*-map from *P*(*X*_*τ*_, ∗) into *P*(*O*_*ρ*_, ∘), denoted by $\hat{f}$. Then for every *c*-bitopological semigroup (*X*_*τ*_12__, ∗, *X*), $\hat{X}$ is an* S*-map from *P*(*X*_*τ*_2__, ∗) into *P*(*X*_*τ*_1__, ∗). That is, the triple $\left(P{\left(X\right)}_{\tau_{12}^{c}},p(*),\hat{X}\right)$ is an *c*-bitopological semigroup where *τ*_1_^*c*^ and *τ*_2_^*c*^ are compact-open topologies on *P*(*X*) which are induced by *τ*_1_ and *τ*_2_, respectively. The shorter notion for this c-bitopological semigroup will be *P*(*X*_*τ*_12__, ∗, *X*).

For a map *f* : *X*_*τ*_ → *O*_*ρ*_, by Δ(*f*), we mean the set

(13) $$\begin{array}{c} \Delta \left(f\right)=\left\{\left(x,\alpha \right)\in X_{\tau }\times P\left(O_{\rho }\right)\;|\;\alpha \left(0\right)=f\left(x\right)\right\}. \end{array}$$

**Proposition 8 .**

Let *f* : (*X*_*τ*_, ∗)→(*O*_*ρ*_, ∘) be an* S*-map. Then (Δ(*f*)|_*τ*×*ρ*^*c*^_, ∗ × *p*(∘)) is an* S*-subspace of an* S*-space ((*X* × *P*(*O*))_*τ*×*ρ*^*c*^_, ∗ × *p*(∘)), where *ρ* ^*c*^ is a compact-open topology on *P*(*O*) which is induced by *ρ*.

**Proof**

It is clear that Δ(*f*)|_*τ*×*ρ*^*c*^_ is a subspace of a space (*X* × *P*(*O*))_*τ*×*ρ*^*c*^_. We observe that, for all (*x*, *α*), (*x*′, *α*′) ∈ Δ(*f*),

(14) $$\begin{array}{c} \left(\alpha p\left(\circ \right)\alpha^{\prime }\right)\left(0\right)=\alpha \left(0\right)\circ \alpha^{\prime }\left(0\right)=f\left(x\right)\circ f\left(x^{\prime }\right)=f\left(x*x^{\prime }\right). \end{array}$$

That is,

(15) $$\begin{array}{c} \left(x,\alpha \right)*\times p\left(\circ \right)\left(x^{\prime },\alpha^{\prime }\right)=\left(x*x^{\prime },\alpha p\left(\circ \right)\alpha^{\prime }\right)\in \Delta \left(f\right). \end{array}$$

Hence (Δ(*f*)|_*τ*×*ρ*^*c*^_, ∗ × *p*(∘)) is an* S*-subspace of an* S*-space ((*X* × *P*(*O*))_*τ*×*ρ*^*c*^_, ∗ × *p*(∘)).

In the last theorem, the shorter notion for the* S*-space (Δ(*f*)|_*τ*×*ρ*^*c*^_, ∗ × *p*(∘)) will be Δ(*f*)|_*τ*×*ρ*^*c*^_.

**Definition 9 .**

Let *f*_12_ : (*X*_*τ*_12__, ∗, *X*)→(*O*_*ρ*_, ∘) be an *c*-map. An* S*-map

(16) $$\begin{array}{c} L:\Delta \left(f_{2}\right){|}_{\tau_{2}\times \rho^{c}}⟶P\left(X_{\tau_{1}},*\right) \end{array}$$

from an* S*-space Δ(*f*_2_)|_*τ*_2_×*ρ*^*c*^_ into *P*(*X*_*τ*_1__, ∗) is called an* c-lifting function* for an *c*-map *f*_12_ provided *L* satisfies the following:

1. *L*(*x*, *α*)(0) = *X*(*x*) for all (*x*, *α*) ∈ Δ(*f*_2_);
2. *f*_1_∘*L*(*x*, *α*) = *α* for all (*x*, *α*) ∈ Δ(*f*_2_).

And *λ*_*f*_ will be denoted to *c*-lifting function for an *c*-map *f*_12_, if it exists.

**Example 10 .**

Let (*X*_*τ*_12__, ∗, *X*) be an *c*-bitopological semigroup. For every* S*-space (*O*_*ρ*_, ∘), the *Sp*-map

(17) $$\begin{array}{c} f_{12}=\left(f,f\right):\left({\left(X\times O\right)}_{\tau_{12}\times \rho },*\times \circ ,X\times id\right)⟶\left(O_{\rho },\circ \right) \end{array}$$

is an *c*-map, where *f*(*x*, *y*) = *y* for all *x* ∈ *X*, *y* ∈ *O*. Note that

(18) $$\begin{array}{c} \left[f\circ \left(X\times \text{id}\right)\right]\left(x,y\right)=f\left(X\left(x\right),y\right)=y=f\left(x,y\right) \end{array}$$

for all *x* ∈ *X*, *y* ∈ *O*. This *c*-map has an *c*-lifting function

(19) $$\begin{array}{c} \lambda_{f}:\Delta \left(f\right){|}_{(\tau_{2}\times \rho )\times \rho^{c}}⟶P\left({\left(X\times O\right)}_{\tau_{1}\times \rho },\tau_{1}\times \circ \right) \end{array}$$

which is given by

(20) λf((x,b),α)(t)=(X(x),α(t)) ∀((x,b),α)∈Δ(f),  t∈I.

Note that

(21) λf((x,b),α)(0)=(X(x),α(0))=(X(x),f(x,b))=(X(x),b)=(X×id)(x,b),[f∘λf((x,b),α)](t)=f(X(x),α(t))=α(t)

for all ((*x*, *b*), *α*) ∈ Δ(*f*), *t* ∈ *I*.

The following theorem clarifies the existence property for *c*-lifting function in *C*_*ℵ*_-fibration theory. That is, it clarifies that the existence of *c*-lifting function for any *C*_*ℵ*_-fibration is necessary and sufficient condition.

**Theorem 11 .**

An *c*-map *f*_12_ : (*X*_*τ*_12__, ∗, *X*)→(*O*_*ρ*_, ∘) is an *C*_*ℵ*_-fibration if and only if there exists an *c*-lifting function for *f*_12_.

**Proof**

Suppose that *f*_12_ is an *C*_*ℵ*_-fibration. Take Δ(*f*_2_)|_*τ*_2_×*ρ*^*c*^_ ∈ *ℵ*. Define two* S*-maps

(22) $$\begin{array}{c} {g:\Delta \left(f_{2}\right)|}_{\tau_{2}\times \rho^{c}}⟶\left(X_{\tau_{2}},*\right),\\ {G:\Delta (f_{2})|}_{\tau_{2}\times \rho^{c}}⟶P\left(O_{\rho },\circ \right) \end{array}$$

by *g*(*x*, *α*) = *x* and *G*(*x*, *α*) = *α* for all (*x*, *α*) ∈ Δ(*f*_2_), respectively. We observe that

(23) $$\begin{array}{c} G\left(x,\alpha \right)\left(0\right)=\alpha \left(0\right)=f_{2}\left(x\right)=\left(f_{2}\circ g\right)\left(x,\alpha \right). \end{array}$$

Since *f*_12_ is an *C*_*ℵ*_-fibration, then there exists an* S*-homotopy *H* : Δ(*f*_2_)|_*τ*_2_×*ρ*^*c*^_ → *P*(*X*_*τ*_1__, ∗) such that *H*_0_ = *X*∘*g* and *f*_1_∘*H*_*t*_ = *G*_*t*_ for all *t* ∈ *I*. Define an* S*-map

(24) $$\begin{array}{c} {\lambda_{f}:\Delta (f_{2})|}_{\tau_{2}\times \rho^{c}}⟶P\left(X_{\tau_{1}},*\right) \end{array}$$

by

(25) $$\begin{array}{c} \lambda_{f}\left(x,\alpha \right)\left(t\right)=H\left(x,\alpha \right)\left(t\right)\;\forall \left(x,\alpha \right)\in \Delta \left(f_{2}\right). \end{array}$$

We observe that, for all (*x*, *α*) ∈ Δ(*f*_2_),

(26) $$\begin{array}{c} \lambda_{f}\left(x,\alpha \right)\left(0\right)=H\left(x,\alpha \right)\left(0\right)=\left(X\circ g\right)\left(x,\alpha \right)=X\left(x\right)\\ f_{1}\circ \lambda_{f}\left(x,\alpha \right)=f_{1}\circ H\left(x,\alpha \right)=G\left(x,\alpha \right)=\alpha . \end{array}$$

That is, *λ*_*f*_ is an *c*-lifting function for *f*_12_.

Conversely, suppose that there exists an *c*-lifting function *λ*_*f*_ for *f*_12_. Let (*Y*_*ω*_, ⋆) ∈ *ℵ* and let *g* : (*Y*_*ω*_, ⋆)→(*X*_*τ*_2__, ∗) and *G* : (*Y*_*ω*_, ⋆) → *P*(*O*_*ρ*_, ∘) be two given* S*-maps with *G*_0_ = *f*_2_∘*g*. Define an* S*-homotopy *H* : (*Y*_*ω*_, ⋆) → *P*(*X*_*τ*_1__, ∗) by

(27) $$\begin{array}{c} H\left(y\right)\left(t\right)=\lambda_{f}\left[g\left(y\right),G\left(y\right)\right]\left(t\right)\;\forall y\in Y,\;\;t\in I. \end{array}$$

We observe that

(28) $$\begin{array}{c} H\left(y\right)\left(0\right)=\lambda_{f}\left[g\left(y\right),G\left(y\right)\right]\left(0\right)=\left(X\circ g\right)\left(y\right),\\ f_{1}\left[H\left(y\right)\left(t\right)\right]=f_{1}\left[\lambda_{f}\left[g\left(y\right),G\left(y\right)\right]\left(t\right)\right]=G\left(y\right)\left(t\right) \end{array}$$

for all *y* ∈ *Y*, *t* ∈ *I*. That is, *H*_0_ = *X*∘*g* and *f*_1_∘*H*_*t*_ = *G*_*t*_ for all *t* ∈ *I*. Hence *f*_12_ is an *C*_*ℵ*_-fibration.

**Theorem 12 .**

Let *f*_12_ : (*X*_*τ*_12__, ∗, *X*)→(*O*_*ρ*_, ∘) be an *C*_*ℵ*_-fibration. Then the c-map

(29) $$\begin{array}{c} {\hat{f}}_{12}=\left({\hat{f}}_{1},{\hat{f}}_{2}\right):P\left(X_{\tau_{12}},*,X\right)⟶P\left(O_{\rho },\circ \right) \end{array}$$

is an *C*_*ℵ*_-fibration.

**Proof**

Since *f*_12_ is an *C*_*ℵ*_-fibration, then there exists *c*-lifting function

(30) $$\begin{array}{c} {\lambda_{f}:\Delta (f_{2})|}_{\tau_{2}\times \rho^{c}}⟶P\left(X_{\tau_{1}},*\right) \end{array}$$

for *f*_12_ such that

(31) $$\begin{array}{c} \lambda_{f}\left(x,\alpha \right)\left(0\right)=X\left(x\right),\\ f_{1}\circ \lambda_{f}\left(x,\alpha \right)=\alpha \end{array}$$

for all (*x*, *α*) ∈ Δ(*f*_2_). Let (*Y*_*ω*_, ⋆) ∈ *ℵ* and let *g* : (*Y*_*ω*_, ⋆) → *P*(*X*_*τ*_2__, ∗) and *G* : (*Y*_*ω*_, ⋆) → *P*[*P*(*O*)_*ρ*^*c*^_, *p*(∘)] be two given* S*-maps with

(32) $$\begin{array}{c} \left[G\left(y\right)\left(s\right)\right]\left(0\right)=\left({\hat{f}}_{2}\circ g\right)\left(y\right)\left(s\right) \end{array}$$

for all *y* ∈ *Y*, *s* ∈ *I*, where *ρ* ^*c*^ is a compact-open topology on *P*(*O*) which is induced by *ρ*. Define an* S*-homotopy *H* : (*Y*_*ω*_, ⋆) → *P*[*P*(*X*)_*τ*_1_^*c*^_, *p*(∗)] by

(33) [H(y)(s)](t)=λf[g(y)(s),G(y)(s)](t)∀y∈Y,  s,t∈I.

We observe that

(34) [H(y)(s)](0)=λf[g(y)(s),G(y)(s)](0)=X[g(y)(s)]=(X^∘g)(y)(s),(f^1∘Hr)(y)(t)=(f1∘λf[g(y)(s),G(y)(s)])(t)=[G(y)(s)](t),

for all *y* ∈ *Y*, *s*, *t* ∈ *I*. That is, $H_{0}=\hat{X}\circ g$ and ${\hat{f}}_{1}\circ H_{s}=G_{s}$ for all *s* ∈ *I*. Hence ${\hat{f}}_{12}$ is an *C*_*ℵ*_-fibration.

An *c*-lifting function *λ*_*f*_ is called* regular* if for every *x* ∈ *X*_*τ*_2__, $\lambda_{f}(x,f_{2}\circ \tilde{x})=\tilde{X(x)}$, where $\tilde{x}$ is the constant path in *X*_*τ*_2__ (i.e., $\tilde{x}(t)=x$), similar for $\tilde{X(x)}$. An *C*_*ℵ*_-fibration *f*_12_ is called* regular* if it has regular c-lifting function.

**Example 13 .**

In Example 10, the *c*-lifting function *λ*_*f*_ which is given by

(35) λf((x,b),α)(t)=(X(x),α(t)) ∀((x,b),α)∈Δ(f),  t∈I

is regular. Note that, for every (*x*, *b*)∈(*X* × *O*)_*τ*_2__,

(36) λf((x,b),f2∘(x,b)~)(t)=(X(x),(f2∘(x,b)~)(t))=(X(x),f2(x,b))=(X(x),b)=(X×id)(x,b) =(X×id)(x,b)~(t)

for all *t* ∈ *I*.

The following theorem is an analogue of results of Fadell in Hurewicz fibration theory [7].

**Theorem 14 .**

Let *f*_12_ : (*X*_*τ*_12__, ∗, *X*)→(*O*_*ρ*_, ∘) be a regular *C*_*ℵ*_-fibration and let

(37) $$\begin{array}{c} M_{i}:P\left(X_{\tau_{i}},*\right)⟶{\Delta (f_{i})|}_{\tau_{i}\times \rho^{c}} \end{array}$$

be an* S*-map defined by *M*_*i*_(*α*) = (*α*(0), *f*_*i*_∘*α*) for all *α* ∈ *P*(*X*_*τ*_*i*__) where *i* = 1,2. Then

(1) *M*_1_∘*λ*_*f*_ = *X* × id|_Δ(*f*_2_)_;
(2) $\lambda_{f}\circ M_{2}\;{≃}_{s}\;\hat{X}$ preserving projection. That is, there is an* S*-homotopy
  (38) $$\begin{array}{c} H:P\left(X_{\tau_{2}},*\right)⟶P\left[P{\left(X\right)}_{\tau_{1}^{c}},p\left(*\right)\right] \end{array}$$
  between two* S*-maps *λ*_*f*_∘*M*_2_ and $\hat{X}$ such that *f*_1_[(*H*(*α*)(*s*))(*t*)] = *f*_2_(*α*(*t*)) for all *t*, *s* ∈ *I*, *α* ∈ *P*(*X*_*τ*_2__).

**Proof**

For the first part, we observe that, for every (*x*, *α*) ∈ Δ(*f*_2_),

(39) (M1∘λf)(x,α)=M1[λf(x,α)]=(λf(x,α)(0),f1∘λf(x,α))=(X(x),α)=(X×id)(x,α).

That is, *M*_1_∘*λ*_*f*_ = *X* × id|_Δ(*f*_2_)_.

For the second part, for *α* ∈ *P*(*X*_*τ*_2__) and *s* ∈ *I*, define a path *β* ^1−*s*^ ∈ *P*(*O*_*ρ*_) by

(40) $$\begin{array}{c} \beta^{1-s}\left(t\right)=\{\begin{array}{cc} f_{2}\left(\alpha \left(s+t\right)\right), & \text{for}\;\;0\leq t\leq 1-s,\\ f_{2}\left(\alpha \left(1\right)\right), & \text{for}\;\;1-s\leq t\leq 1. \end{array} \end{array}$$

By the regularity of *λ*_*f*_, we can define an* S*-homotopy *H* : *P*(*X*_*τ*_2__, ∗) → *P*[*P*(*X*)_*τ*_1_^*c*^_, *p*(∗)] by

(41) $$\begin{array}{c} \left[H\left(\alpha \right)\left(s\right)\right]\left(t\right)=\{\begin{array}{cc} X\left[\alpha \left(t\right)\right], & \text{for}\;\;0\leq t\leq s,\\ \lambda_{f}\left(\alpha \left(s\right),\beta^{1-s}\right)\left(t-s\right), & \text{for}\;\;s\leq t\leq 1, \end{array} \end{array}$$

for all *s* ∈ *I*, *α* ∈ *P*(*X*_*τ*_2__). Then

(42) [H(α)(0)](t)=λf(α(0),β1)(t)=λf(α(0),β)(t)=λf(α(0),f2∘α)(t)=(λf∘M2)(α)(t),[H(α)(1)](t)=X[α(t)]=X^(α)(t)

for all *α* ∈ *P*(*X*_*τ*_2__), *t* ∈ *I*. That is, $\lambda_{f}\circ M_{2}\;{≃}_{s}\;\hat{X}$. Also we get that

(43) [f1∘(H(α)(s))](t)  ={f1(X[α(t)]),for  0≤t≤s,f1[λf(α(s),β1−s)(t−s)],for  s≤t≤1;  ={f2(α(t)),for  0≤t≤s,β1−s(t−s),for  s≤t≤1;  ={f2(α(t)),for  0≤t≤s,f2(α(s+t−s)),for  s≤t≤1;  ={f2(α(t)),for  0≤t≤s,f2(α(t)),for  s≤t≤1;  =f2(α(t)),

for all *s*, *t* ∈ *I*, *α* ∈ *P*(*X*_*τ*_2__). Hence $\lambda_{f}\circ M_{2}\;{≃}_{s}\;\hat{X}$ preserving projection.

## 5. Approximate Fibrations

Coram and Duvall [2] introduced approximate fibrations as a generalization of cell-like maps [3] and showed that the uniform limit of a sequence of Hurewicz fibrations is an approximate fibration. A map *f* : *X*_*τ*_ → *O*_*ρ*_ of compact metrizable spaces *X*_*τ*_ and *O*_*ρ*_ is called an* approximate fibration* if, for every space *Y*_*ω*_ and for given *ϵ* > 0, there exists *δ* > 0 such that whenever *g* : *Y*_*ω*_ → *X*_*τ*_ and *H* : *Y*_*ω*_ × *I* → *O*_*ρ*_ are maps with *d*[*H*(*y*, 0), (*f*∘*g*)(*y*)] < *δ*, then there is homotopy *G* : *Y*_*ω*_ × *I* → *X*_*τ*_ such that *G*_0_ = *g* and

(44) $$\begin{array}{c} d\left[H\left(y,t\right),\left(f\circ G\right)\left(y,t\right)\right]<\epsilon \;\forall y\in Y,\;t\in I. \end{array}$$

One notable exception is that the pullback of approximate fibration need not be an approximate fibration.

The following theorem shows the role of the *C*_*N*_*π*__-fibration property in inducing an approximate fibration property.

For an* S*-map *f* : (*X*_*τ*_, *π*)→(*O*_*ρ*_, *π*) with metrizable spaces *X*_*τ*_ and *O*_*ρ*_, by *d*_*τ*_ and *d*_*ρ*_, we mean the metric functions on *X* and *O*, respectively; by *X* × *O* we mean the product metrizable space of *X*_*τ*_ and *O*_*ρ*_ with a metric function

(45) $$\begin{array}{c} d\left(\left(x,b\right),\left(x^{\prime },b^{\prime }\right)\right)=\max\left\{d_{\tau }\left(x,x^{\prime }\right),d_{\rho }\left(b,b^{\prime }\right)\right\}; \end{array}$$

by *G* ^*f*^ we mean the graph of *f* (i.e., *G* ^*f*^ = {(*x*, *f*(*x*)) : *x* ∈ *X*}) which is an* S*-subspace of ((*X* × *O*)_*τ*×*ρ*_, *π*); for a positive integer *n* > 0, by *G* ^*n*^(*f*), we mean the (1/*n*)-neighborhood of *G* ^*f*^ in a metrizable space *X* × *O* which is also* S*-subspace of ((*X* × *O*)_*τ*×*ρ*_, *π*).

**Theorem 15 .**

Let *f* : *X*_*τ*_ → *O*_*ρ*_ be a map with compact metrizable spaces *X*_*τ*_ and *O*_*ρ*_. Then *f* is an approximate fibration if and only if, for every positive integer *n* > 0, there exists a positive integer *m* ≥ *n* such that the* S*-map *f*_*n*_ : (*G* ^*n*^(*f*), *π*)→(*O*_*ρ*_, *π*) has the *C*_*N*_*π*__-fibration property by the inclusion* S*-map *I*_*m*_^*n*^ : (*G* ^*m*^(*f*), *π*)→(*G* ^*n*^(*f*), *π*), where *f*_*n*_(*x*, *b*) = *b* for all (*x*, *b*) ∈ *G* ^*n*^(*f*).

**Proof**

Let *n* be any positive integer. For *ϵ* = 1/*n* > 0, let *δ* be given in the definition of approximate fibration. Since *δ*/2 > 0 and *f* is a continuous function, then let *δ*′ be chosen such that if *x*, *x*′ ∈ *X* and *d*_*τ*_(*x*, *x*′) < *δ*′, then *d*_*ρ*_(*f*(*x*), *f*(*x*′)) < *ϵ*/2. Choose a positive integer *m*⩾*n*, such that 1/*m* ≤ *δ*′, *δ*/2.

Now let (*Y*_*ω*_, *π*) ∈ *N*_*π*_ and let *g* : (*Y*_*ω*_, *π*)→(*G* ^*m*^(*f*), *π*) and *G* : (*Y*_*ω*_, *π*) → *P*(*O*_*ρ*_, *π*) be two given* S*-maps with *G*_0_ = *f*_*n*_∘(*I*_*m*_^*n*^∘*g*). Define a map *g*′ : *Y*_*ω*_ → *X*_*τ*_ by *g*′(*y*) = *J*_1_[*g*(*y*)] and a homotopy *G*′ : *Y*_*ω*_ × *I* → *O*_*ρ*_ by *G*′(*y*, *t*) = *G*(*y*)(*t*) for all *y* ∈ *Y* and *t* ∈ *I*. We get that *g*(*y*) = (*g*′(*y*), *G*′(*y*, 0)) for all *y* ∈ *Y*. Since *g*(*y*) ∈ *G* ^*m*^(*f*), then there exists *x* ∈ *X* such that

(46) $$\begin{array}{c} d\left[\left(x,f\left(x\right)\right),g\left(y\right)\right]<\frac{1}{m}. \end{array}$$

Then

(47) $$\begin{array}{c} d_{\tau }\left(x,g^{\prime }\left(y\right)\right)<\frac{1}{m}⩽\delta^{\prime },\\ d_{\rho }\left(f\left(x\right),G^{\prime }\left(y,0\right)\right)<\frac{1}{m}⩽\frac{\delta }{2},\\ d_{\rho }\left(f\left(x\right),f\left(g^{\prime }\left(y\right)\right)\right)<\frac{1}{m}⩽\frac{\delta }{2} \end{array}$$

for all *y* ∈ *Y*. This implies

(48) dρ(f(g′(y)),G′(y,0))  ⩽dρ(f(g′(y)),f(x))+dρ(f(x),G′(y,0))  <δ

for all *y* ∈ *Y*. Hence, since *f* is an approximate fibration, there exists a homotopy *H*′ : *Y*_*ω*_ × *I* → *X*_*τ*_ such that *H*_0_′ = *g*′ and

(49) $$\begin{array}{c} d_{\tau }\left(G^{\prime }\left(y,t\right),\left(f\circ H_{t}^{\prime }\right)\left(y\right)\right)<\epsilon \end{array}$$

for all *y* ∈ *Y*, *t* ∈ *I*. Define an* S*-homotopy *H* : (*Y*_*ω*_, *π*) → *P*(*G* ^*n*^(*f*), *π*) by

(50) $$\begin{array}{c} H\left(y\right)\left(t\right)=\left(H^{\prime }\left(y,t\right),G\left(y\right)\left(t\right)\right)\;\forall y\in Y,\;\;t\in I. \end{array}$$

Then we get that

(51) H(y)(0)=(H′(y,0),G(y)(0))=(g′(y),G(y)(0))=g(y)=(Imn∘g)(y)

for all *y* ∈ *Y* and *f*_*n*_∘*H*_*t*_ = *G*_*t*_ for all *t* ∈ *I*. Hence *f*_*n*_ has the *C*_*N*_*π*__-fibration property by *I*_*m*_^*n*^.

Conversely, let *ϵ* > 0 be given. Since *f* is a continuous function, then let *δ*′ be chosen such that if *x*, *x*′ ∈ *X* and *d*_*τ*_(*x*, *x*′) < *δ*′, then *d*_*ρ*_(*f*(*x*), *f*(*x*′)) < *ϵ*/2. Choose a positive integer *n* > 0 such that 1/*n* ≤ *δ*′, *ϵ*/2. By hypothesis, there exists a positive integer *m* ≥ *n* such that *f*_*n*_ has the *C*_*N*_*π*__-fibration property by *I*_*m*_^*n*^.

Take *δ* = 1/*m*. Let *Y*_*ω*_ be any space and let *g* : *Y*_*ω*_ → *X*_*τ*_ and *G* : *Y*_*ω*_ × *I* → *O*_*ρ*_ be two given maps with

(52) $$\begin{array}{c} d_{\rho }\left[G\left(y,0\right),\left(f\circ g\right)\left(y\right)\right]<\delta \end{array}$$

for all *y* ∈ *Y*. Define an* S*-map *g*′ : (*Y*_*ω*_, *π*)→(*G* ^*m*^(*f*), *π*) by *g*′(*y*) = (*g*(*y*), *G*(*y*, 0)) and an* S*-homotopy *G*′ : (*Y*_*ω*_, *π*) → *P*(*O*_*ρ*_, *ı*) by *G*′(*y*)(*t*) = *G*(*y*, *t*) for all *y* ∈ *Y* and *t* ∈ *I*. Since *G*_0_′ = *f*_*n*_∘(*I*_*m*_^*n*^∘*g*′), then there exists an* S*-homotopy *F* : (*Y*_*ω*_, *π*) → *P*(*G* ^*n*^(*f*), *π*) such that *F*_0_ = *I*_*m*_^*n*^∘*g*′ and *f*_*n*_∘*F*_*t*_ = *G*_*t*_′ for all *t* ∈ *I*. By the last part, we can define a homotopy *H* : *Y*_*ω*_ × *I* → *X*_*τ*_ by

(53) $$\begin{array}{c} H\left(y,t\right)=J_{1}\left[F\left(y\right)\left(t\right)\right]\;\forall y\in Y,\;\;t\in I. \end{array}$$

We get that *F*(*y*)(*t*) = (*H*(*y*, *t*), *G*(*y*, *t*)). Since *F*(*y*)(*t*) ∈ *G* ^*n*^(*f*), then there exists *x* ∈ *X* such that

(54) $$\begin{array}{c} d\left[\left(x,f\left(x\right)\right),F\left(y\right)\left(t\right)\right]<\frac{1}{n}. \end{array}$$

Then

(55) $$\begin{array}{c} d_{\tau }\left(x,H\left(y,t\right)\right)<\frac{1}{n}⩽\delta^{\prime },\;d_{\rho }\left(f\left(x\right),G\left(y,t\right)\right)<\frac{1}{n}⩽\frac{\epsilon }{2},\\ d_{\rho }\left[f\left(x\right),f\left(H\left(y,t\right)\right)\right]<\frac{1}{n}⩽\frac{\epsilon }{2}. \end{array}$$

This implies

(56) dρ[G(y,t),f(H(y,t))]  ⩽dρ[f(H(y,t)),f(x)]+dρ(f(x),G(y,t))<ϵ

for all *y* ∈ *Y*, *t* ∈ *I*. Hence *f* is an approximate fibration.
